# Image-based characterization of thrombus formation in time-lapse DIC microscopy

**DOI:** 10.1016/j.media.2012.02.002

**Published:** 2012-05

**Authors:** Nicolas Brieu, Nassir Navab, Jovana Serbanovic-Canic, Willem H. Ouwehand, Derek L. Stemple, Ana Cvejic, Martin Groher

**Affiliations:** a Computer Aided Medical Procedures, Technische Universität München (TUM), Garching bei München 85748, Germany; b Department of Hematology, University of Cambridge & NHS Blood and Transplant, Cambridge CB2 0PT, United Kingdom; c The Wellcome Trust Sanger Institute, Hinxton, Cambridge CB10 1SA, United Kingdom

**Keywords:** Time-lapse microscopy, DIC microscopy, Motion-segmentation, Dynamic texture, Tracking

## Abstract

The characterization of thrombus formation in time-lapse DIC microscopy is of increased interest for identifying genes which account for atherothrombosis and coronary artery diseases (CADs). In particular, we are interested in large-scale studies on zebrafish, which result in large amount of data, and require automatic processing. In this work, we present an image-based solution for the automatized extraction of parameters quantifying the temporal development of thrombotic plugs. Our system is based on the joint segmentation of thrombotic and aortic regions over time. This task is made difficult by the low contrast and the high dynamic conditions observed *in vivo* DIC microscopic scenes. Our key idea is to perform this segmentation by distinguishing the different motion patterns in image time series rather than by solving standard image segmentation tasks in each image frame. Thus, we are able to compensate for the poor imaging conditions. We model motion patterns by energies based on the idea of dynamic textures, and regularize the model by two prior energies on the shape of the aortic region and on the topological relationship between the thrombus and the aorta. We demonstrate the performance of our segmentation algorithm by qualitative and quantitative experiments on synthetic examples as well as on real *in vivo* microscopic sequences.

## Graphical abstract

**Graphical abstract:**

**Figure:**
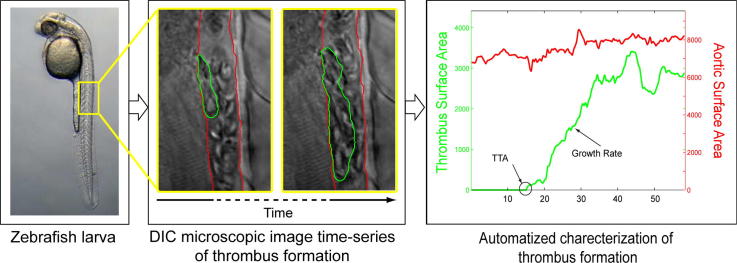

**Highlights:**

► Automatized characterization of thrombus formation in time-lapse microscopy. ► Novel energy model for segmentation of multiple dynamic regions. ► Novel algorithm for the joint segmentation of thrombus and aortic regions. ► Exhaustive validation on synthetic and real microscopic data.

## 1 Introduction

Thrombosis denotes the abnormal aggregation of blood cells which occurs after a blood vessel injury and which eventually leads to the partial or complete obstruction of the blood circulation (Furie and Furie, 2005). This malfunction plays a major role in the development of coronary artery diseases (CADs), the leading cause of death in the developed world. In addition to the identification of environmental risk factors such as smoking, obesity, and physical inactivity, the identification of possible genetic disorders is becoming increasingly important to improve the diagnosis and the therapy of CADs. Linking functional defects in thrombus formation with underlying genetic disorders requires large-scale statistical *in vivo* studies on thrombus formation (O’Connor et al., 2009). The increased use of zebrafish has been recently prompted by their high fecundity and their rapid *ex vivo* development. Some of their advantages over other animal models also include the molecular and genetic similarity of their coagulation process with the coagulation process of humans, the existence of an efficient and rapid technology to knockdown gene expression, and their optical transparency. This makes the *in vivo* visualization of thrombus formation possible even without sophisticated equipment or invasive surgery. We refer the reader to (Jagadeeswaran et al., 2005; Carradice and Lieschke, 2008; Lieschke and Currie, 2007; Nasevicius and Ekker, 2000; O’Connor et al., 2009) for further details on the vascular injury model in zebrafish.

**Fig. 1:**
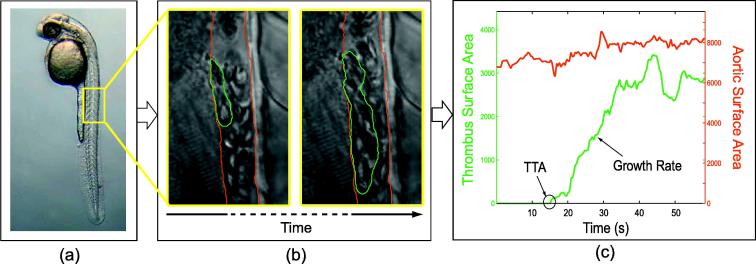
We study thrombus formation in zebrafish larvae (a). The accumulation of blood cells is observed *in vivo* via a CCD camera mounted on a DIC microscope, resulting in a huge amount of image data (b). The segmentation of the thrombus (green) and vessel (red) regions in time lead to the derivation of measures characterizing the temporal development of the thrombus (c). (For interpretation of the references to colour in this figure legend, the reader is referred to the web version of this article.)

We study the formation of thrombus on zebrafish larvae with the following experimental setup (O’Connor et al., 2009). The aggregation of blood cells is provoked by artificially injuring the wall of the caudal aorta with a laser pulse. Images of the scene are recorded by a CCD camera mounted on a Differential Interference Contrast (DIC) microscope. In the current state of the system, biologists manually derive measures such as the time to attachment (TTA) of the first blood cell to the aortic wall and the thrombus surface area (TSA). These measures characterize the formation of the thrombus in time. The less influence a candidate gene has on the formation of the thrombus, the more this experiment has to be repeated to gain a sufficient level of sensitivity, and the more crucial an automatized solution becomes. In this work, and as illustrated in Fig. 1, we propose to derive the aforementioned measures from the segmentation of the thrombus and of the aortic regions over time.

The poor quality of *in vivo* DIC microscopic images makes such segmentation difficult to obtain automatically. The low contrast between the thrombus, the uncoagulated vessel, and the background regions hampers the computation of reliable intensity-based segmentation cues. The circulating blood cells in the vessel and the static structures in the background introduce a high number of disrupting edges, which make the derivation of discriminative gradient-based features and the use of common gradient-based approaches hardly possible.

To overcome these difficulties, the key idea of this work is to replace image segmentation in single-frame by motion segmentation in sub-sequences of images. More precisely, we propose to characterize the variations of the motion patterns both in space and in time. Motion is modeled using the concept of dynamic textures (DTs) (Doretto et al., 2003a). We define the segmentation of the thrombus and of the vessel regions at a given frame as an energy minimization problem. A set of likelihood and prior energies are introduced to account for the three following observations:

(1) The thrombus, the uncoagulated vessel, and the background are defined by distinct motion patterns: the thrombus is moving slowly and synchronously with the heart beat, the uncoagulated vessel witnesses the fast and chaotic circulation of blood cells, and the background remains nearly static. We guarantee the spatial homogeneity of the motion patterns in each region by introducing a DT-based *motion-segmentation* likelihood. The main advantage of this novel likelihood in comparison to prior works on DT segmentation is its capacity to handle multiple dynamic regions while maintaining the simplicity of the closed-formed solution for the estimation of the motion patterns. Our solution is based on the approximation of the normal distribution in the manifold of the DT models (Pennec, 2006).
(2) The thrombus is the only region which does not exist at the initial state of the video sequence, i.e. before the attachment of the first blood cell. This observation leads to the modeling of the thrombus as an abnormal event in comparison to the circulation of blood cells in the uncoagulated vessel. Because of the low contrast observed in *in vivo* microscopic scenes, we characterize such abnormal events by a significant change of the motion patterns in time. To this end we introduce a DT-based *event-detection* likelihood.
(3) The two aforementioned likelihoods can be locally disrupted by the slow circulation of blood cells in the collateral vessels and by local non-rigid movement of the epithelium. From the observations that the vessel resembles a tubular shape and that the thrombus is included in the vessel, we introduce a shape prior and a topological energy. These quantities constrain the model sufficiently to achieve accurate segmentation results.

The energy obtained by combining (1), (2), and (3) is minimized using multiphase level-sets (Vese and Chan, 2002). As shown by qualitative and quantitative experiments on synthetic examples and real microscopic data, the introduced level-set framework leads to the accurate segmentation of the biological scene. By successively applying our algorithm over time, we are then able to successfully track the thrombus and the vessel regions and to accurately retrieve the temporal variations of the thrombus surface area.

Our contribution is twofold. First, from an application point of view, we develop an innovative solution for the automatized characterization of thrombus formation from DIC microscopic image time-series. This solution is based on the joint segmentation of thrombus and aortic regions in time. Second, from a methodological point of view, we introduce a simple yet accurate energy model for the segmentation of multiple dynamic regions (1). The resulting ability of our algorithm to analyze complex dynamic scene makes it of general interest for the medical imaging and computer vision communities.

### 1.1 Related work

Our application is related to the field of microscopic image analysis and more precisely to the segmentation and the tracking of cells. We review related approaches in Section 1.1.1. In our scenario, the unreliability of segmentation methods based on the analysis of single-frames motivates the analysis of the motion patterns, and more specifically the use of dynamic textures. Therefore, we also review previous motion-segmentation methods based on dynamic textures in Section 1.1.2.

#### 1.1.1 Microscopic image analysis

Some of the main applications of microscopic image analysis include cell lineage construction (Huh et al., 2011; Li et al., 2008), cell segmentation and tracking (Cheng and Rajapakse, 2009; Dong et al., 2005, 2007; Dzyubachyk et al., 2010; Eden et al., 2005; Kamoun et al., 2008; Mukherjee et al., 2004; Nguyen et al., 2011; Padfield et al., 2009, 2011; Quelhas et al., 2010; Ray et al., 2002), cell recognition (Long et al., 2005, 2008), and vessel segmentation (Tang and Acton, 2004; Schmugge et al., 2008). The low contrast of DIC microscopic images makes the segmentation and detection methods commonly employed in fluorescence microscopy hardly applicable in our case (Cheng and Rajapakse, 2009; Dzyubachyk et al., 2010; Kamoun et al., 2008; Nguyen et al., 2011; Padfield et al., 2009; Padfield et al., 2011; Quelhas et al., 2010). Because of the low contrast conditions also observed in brightfield microscopy, our scenario is more closely related to the problem of *in vivo* tracking of rolling leukocyte presented in (Dong et al., 2005, 2007; Mukherjee et al., 2004; Ray et al., 2002). In these works, segmentation is based on gradient-based parametric active contours (Dong et al., 2005, 2007; Ray et al., 2002) or on intensity-based level-set methods (Mukherjee et al., 2004). However, these approaches are hardly applicable to our scenario because of the necessity to incorporate a prior knowledge on the circular shape of the leukocytes to compensate for the cluttering of the aortic vessel by blood flow-related stream lines. The active contour presented in (Ray et al., 2002) is constrained with an elliptic shape prior. In (Dong et al., 2005), a more advanced constraint, the GICOV coefficient, ensures the consistency of the gradient magnitude along the leukocyte boundary. In (Mukherjee et al., 2004), the circular shape of the leukocytes is accounted with a slenderness measure. In our case, the accumulation of blood cells results in a high variability in shape and in size of the thrombus region in time. As such, the priors presented in (Dong et al., 2005, 2007; Mukherjee et al., 2004; Ray et al., 2002) could hardly be employed to help the segmentation of the thrombus region. As showed in (Ersoy and Palaniappan, 2008), the unreliability of the gradient and intensity-related features in time-lapse brightfield microscopy can be successfully compensated by analyzing the motion patterns. The authors introduce a flux tensor to discriminate moving human melanoma cells from a static background. *In vitro* experiments are conducted. The flux tensor faces two main limitations. First, it is restricted to the distinction between moving and stationary pixels. In our scenario, the method developed in (Ersoy and Palaniappan, 2008) would thus most likely fail to discriminate the slowly moving thrombus from the fast circulating blood cells. Furthermore, it relies on the optical flow equation and on the brightness consistency assumption, and would not be robust to the fast and chaotic circulation of the blood cells in the aortic vessel. Compared to (Ersoy and Palaniappan, 2008), we choose to employ the dynamic texture (DT) model of Doretto et al. (2003a) which has been extensively shown to provide reliable motion cues even on such complex scenarios (Chan and Vasconcelos, 2008, 2009; C, 2010; Doretto et al., 2003b; Huang et al., 2007; Vidal et al., 2007). Another motion-based approach is proposed by Eden et al. (2005) to segment rolling leukocytes in brightfield microscopy. It operates in two steps: first the segmentation of the vessel from the background, second the segmentation of the circulating cells using motion, color, and texture information. The key assumption is that the circulating cells are in constant motion. This does not hold in our scenario because of the back-and-forth movement of the thrombus. Compared to (Eden et al., 2005), we propose a more general motion model and only make the assumption of different motion patterns in the different regions.

#### 1.1.2 Dynamic textures

**Fig. 2:**
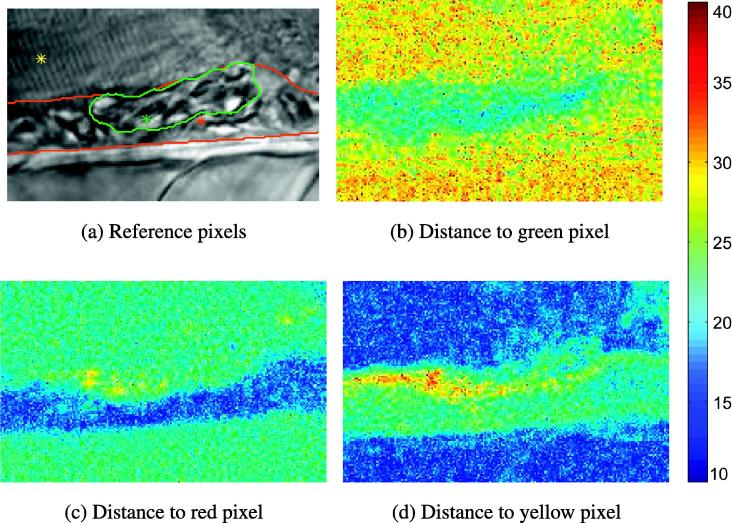
Variability of the Martin distance maps w.r.t the choice of the reference DT-model. The reference DT-model is built from the intensities observed in a spatio-temporal neighborhood (*m* × *τ*) centered at an arbitrary pixel location in the thrombus (b), in the uncoagulated vessel (c), or in the background (d).

Dynamic textures model the variations of the intensity at each pixel with a Linear Dynamic System (LDS) (Doretto et al., 2003a). They permit the representation of both appearance and dynamics in a concise and intuitive formulation. They also benefit from the existence of a closed-form solution for the estimation of their parameters. The first method to address the problem of motion segmentation based on dynamic texture is presented in (Doretto et al., 2003b). It consists of the following steps: (1) the DT models are estimated at each pixel of the image, (2) a spatio-temporal signature map is derived by computing the distance between a reference DT model and the DT models observed at each pixel of the image domain, and (3) the obtained map is segmented using a level-set framework (Chan and Vese, 2001). Fig. 2 displays some signature maps obtained on a sequence of 75 microscopic images with respect to three different reference models. The first reference DT model is built from a pixel in the thrombus, the second reference DT model from a pixel in the vessel, and the third DT model from a pixel in the background. The “single DT model” segmentation method, introduced in (Doretto et al., 2003b), faces two major limitations. First, the signature map depends on the selection of the reference model. Second, two pixels belonging to two different dynamic regions can be characterized by similar distances. It directly follows that (Doretto et al., 2003b) is restricted to the segmentation of less than two dynamic regions and that it could thus hardly be used for the joint segmentation of the thrombus, of the aortic, and of the background regions.

A few approaches have been developed to solve the aforementioned limitations of (Doretto et al., 2003b). Chan and Vasconcelos (2008) extends the single DT model (Doretto et al., 2003a) to a *Mixture of Dynamic Texture* (MDT) model. The intensities are explained as the superposition of co-occurring DT models, whose parameters are estimated using an Expectation Maximization (EM) scheme. The authors further extend the MDT model to the *Layered Dynamic Texture* (LDT) model (Chan and Vasconcelos, 2009) in which the spatial coherence of the DT models is ensured using a Markov random field. The approaches (Chan and Vasconcelos, 2008, 2009) are to the best of our knowledge the only attempts, based on LDSs, to segment scenes with more than two dynamic texture regions. However, the lack of a closed-form solution for the estimation of the model parameters makes these approaches significantly more involved than the methods directly derived from the single DT model (Doretto et al., 2003a). The motion segmentation energy and the multiphase level-set framework introduced in this work retain the advantages of the MDT and LDT algorithms for multiple dynamic texture segmentation while maintaining the closed-form solution, recalled in Section 2.1, for the estimation of single DT models. The aforementioned algorithms for dynamic texture segmentation (Chan and Vasconcelos, 2008, 2009; Doretto et al., 2003b) are derived from the LDS model of Doretto et al. (Doretto et al., 2003a). It is worth noting that Ghoreyshi and Vidal (2007) propose to describe dynamic textures by characterizing the temporal variation of Ising texture descriptors with an autoregressive (AR) model. Segmentation is performed by minimizing, using level-sets, the error between the predicted and the observed texture descriptors. The method (Ghoreyshi and Vidal, 2007) circumvents the need for a reference model and is extended, but not experimentally validated, for the segmentation of multiple dynamic textures in (Vidal et al., 2007). The key advantage of our solution in comparison to (Vidal et al., 2007) is, in addition to its higher accuracy, the possibility to maintain the more commonly employed LDS-based modeling of dynamic textures.

One of the other key applications of dynamic textures is the detection of salient events under low contrast and highly dynamic conditions. These conditions hamper the use of common intensity-based event-detection algorithms (Stauffer and Grimson, 1999; Zivkovic, 2004). Mahadevan et al. (2010) propose to detect abnormal behaviors in a crowded walkway using the MDT model (Chan and Vasconcelos, 2008). In our work, we propose to model the thrombus formation as an abnormal event in comparison to the circulation of blood cells in the uncoagulated aortic vessel. The complexity of the MDT model leads us to introduce an alternative solution, based on the single DT model (Doretto et al., 2003a), to the problem of event-detection under low contrast and dynamic conditions.

## 2 Method

In this overall work, we present an image-based algorithm for the automatized characterization of thrombus formation in DIC-microscopic image sequence. In this section, we focus on the sub-problem of the joint segmentation of the thrombus and aortic regions. We explain how the segmentation is used for tracking the vessel and thrombus regions in Section 3.

Let us denote the complete microscopic video sequence by $S=\{I^{t}\text{,}0⩽t<T\}$. For each time *t*, the aim is to find the correct partition *ω*^*t*^ of the image domain *Ω*^*t*^ into the thrombus $\left(\Omega_{1}^{t}\right)$, the background $\left(\Omega_{2}^{t}\right)$, and the uncoagulated vessel $\left(\Omega_{3}^{t}\right)$ regions. The reunion of the thrombus and of the uncoagulated vessel regions forms the aortic region, $\Omega_{12}^{t}=\Omega_{1}^{t}\cup \Omega_{2}^{t}$. To assess the temporal information necessary to define the two DT-based likelihood energies, we consider the temporal window of *τ* + 1 frames centered at time *t*, $S^{t}=\{I^{t}\text{,}t-\tau /2⩽t^{\prime }⩽t+\tau /2\}$, and the initial temporal window $S^{t_{0}}\;≜\;S^{\tau /2}=\{I^{t}\text{,}0⩽t^{\prime }⩽\tau \}$.

We model the partitioning of the image domain at time *t* given the frames in $S^{t}$ and $S^{t_{0}}$ as the following energy minimization problem:

(1) $$\omega^{t}=\arg\underset{\omega^{t}}{\min}[E(S^{t}\text{,}S^{t_{0}}|\omega^{t})+E(\omega^{t})]\text{.}$$

The likelihood energy $E(S^{t}\text{,}S^{t_{0}}|\omega^{t})$ groups the motion-segmentation *E*_ms_ and the event-detection energies *E*_ed_. Since both energies are based on the concept of dynamic textures and on the derivation of a metric on the dynamic texture manifold, we provide a self-containing description of these two concepts in Section 2.1. The motion-segmentation energy guarantees the homogeneity of the motion-patterns in each region and is presented in Section 2.2. The event-detection energy models the growth of the thrombus in an initially uncoagulated vessel and is detailed in Section 2.3.

The prior energy *E*(*ω*^*t*^) comprises the tubular shape prior energy *E*_tub_ and the topological energy *E*_top_. The tubular shape prior energy, introduced in Section 2.4, constrains the surface of the aortic region and prevents the thrombus and the vessel from leaking into the background. The topological energy guarantees the inclusion of the thrombus in the aortic region. It is introduced in Section 2.5 together with the minimization of Eq. (1) using multiphase level-sets (Vese and Chan, 2002).

### 2.1 Review of the dynamic texture model

In this work, we are interested in the analysis of motion patterns. We thus need a way to describe motion. This can be achieved using the concept of dynamic texture. We will introduce dynamic textures in Section 2.1.1. Furthermore, in order to compare motion, we need a distance measure. For this task, we will employ the Martin distance, which we review in Section 2.1.2. These are the central concepts used in the modeling of the likelihood energies in Sections 2.2 and 2.3.

#### 2.1.1 Dynamic texture model

In this paragraph, we briefly review the dynamic texture (DT) model as introduced by Doretto et al. (2003a). Let us consider the temporal window $S^{t}$ and the square spatial patch of *m* neighboring pixels centered at the pixel **x**. We assume that the intensities $y(t^{\prime })\in R^{m}$ observed in the patch at a time *t*′ ∈ [*t* − *τ*/2, … , *t* + *τ*/2] are related to a set of hidden variables $z(t^{\prime })\in R^{n}$ by the following n-order linear dynamic system:

(2) $$\begin{array}{cc} & z(t^{\prime }+1)=Az(t^{\prime })+v(t^{\prime })\\ & y(t^{\prime })-\overset{¯}{y}=Cz(t^{\prime })+w(t^{\prime })\text{,} \end{array}$$

where $\overset{¯}{y}$ is the mean of **y**(*t*′) over $S^{t}$. The second equation describes the simplification of the pixel intensities in a set of *n* ≪ *m* spatial features. The first equation accounts for the evolution of the features over time. $A\in R^{n\times n}$ and $C\in R^{m\times n}$ are the state-transition and the observation matrices respectively. The Gaussian processes $v(t^{\prime })\propto N(0\text{,}Q)\text{,}\;Q\in R^{n\times n}$, and $w(t^{\prime })\propto N(0\text{,}R)\text{,}\;R\in R^{m\times m}$ account for noise in the hidden and in the observed states.

Given the observations **y**(*t*′) over the temporal window $S^{t}$, the model parameters $D_{x}^{t}=\left\{A\text{,}C\right\}$ can be easily estimated under the assumption that both the observation and the state-transition matrices A and C are independent of the time *t*′ of the transition (Doretto et al., 2003a). The sub-optimal solution reads as follows. Stacking Eq. (2) for the *τ* transitions in $S^{t}$ leads to $Z_{2}^{\tau +1}={\mathrm{AZ}}_{1}^{\tau }+V$ and $Y_{1}^{\tau +1}={\mathrm{CZ}}_{1}^{\tau +1}+W$, where $Z_{p}^{q}=[z(p)\text{,}\ldots \text{,}z(q)]$ and $Y_{p}^{q}=[y(p)-\overset{¯}{y}\text{,}\ldots \text{,}y(q)-\overset{¯}{y}]$. Given the singular value decomposition $Y_{1}^{\tau +1}=U\Sigma V^{⊤}$, assuming the orthogonality of the observation matrix yields to C^=U,Z^1τ+1=ΣV⊤, and A^=Z^2τ+1Z^1τ†, where (.)^†^ denotes the Moore–Penrose pseudo-inverse operator. Please note that the uniqueness of the matrices A^ and C^ is implied by the uniqueness of the singular value decomposition.

#### 2.1.2 Martin distance

We use the Martin distance to compare DT models. The orthogonality constraint on the matrix C yields a non-Euclidean structure for the manifold of the DT models (Saisan et al., 2001). This makes the use of adapted metrics necessary to compare DT models. Fortunately, several appropriate metrics have already been proposed in the literature. The Martin distance (De Cock and De Moor, 2002; Martin, 2002) and the Binet-Cauchy kernel (Vishwanathan et al., 2007) assess the similarity between the observation subspaces of the models, while the Kullback–Leibler divergence (Chan and Vasconcelos, 1992) compares their respective probability distributions. Though the Martin distance is not the actual distance in the manifold, it has been extensively shown to be appropriate to measure distances between motion patterns (Chan and Vasconcelos, 2007; Doretto et al., 2003b; Ravichandran et al., 2009; Saisan et al., 2001). We therefore assume that using this distance is an acceptable approximation to the actual geodesic distance. A further argument which supports this approximation is the extensive use of the Martin distance for non-linear dimensionality reduction of the DT manifold (Ravichandran et al., 2009). The key ideas behind the Martin distance are to neglect the noise processes **w** and **v** and to note that any observed variable generated by a DT model D={A,C} lies in the *n*-dimensional subspace spanned by the columns of the observability matrix O=C^⊤,A^⊤C^⊤,…,(A^n-1)⊤C^⊤⊤∈Rmn×n. The Martin distance between two DT models $D_{1}$ and $D_{2}$ is then defined by the product of the principal angles *θ*_*i*_ between their respective subspaces (De Cock and De Moor, 2002; Martin, 2002):

(3) $$d_{M}^{2}(D_{1}\text{,}D_{2})=-\log\overset{n}{\underset{i=1}{\prod }}\cos^{2}(\theta_{i})=-\log\frac{|O_{1}^{T}O_{2}{|}^{2}}{\left|O_{1}^{T}O_{1}||O_{2}^{T}O_{2}\right|}\text{.}$$

### 2.2 Motion-segmentation likelihood

The purpose of the motion segmentation energy is to ensure the homogeneity of the thrombus $\left(\Omega_{1}^{t}\right)$, of the uncoagulated vessel $\left(\Omega_{2}^{t}\right)$, and of the background $\left(\Omega_{3}^{t}\right)$ regions with respect to the motion patterns, and thus to the DT models. To this end, we extend the statistical approach for region-based segmentation (Cremers et al., 2007) to the segmentation in the manifold of the DT models. The Riemannian structure of this manifold shown by Saisan et al. (2001) makes the use of statistical frameworks for analyzing dynamic scenes difficult. Several clustering algorithms on the Euclidean space such as K-mean (Ravichandran et al., 2009), hierarchical EM (C, 2010), and approximate nearest neighbors (Chaudhry and Ivanov, 2010) have been successfully extended to Riemannian manifolds. Level-set approaches such as the one proposed by Lenglet et al. (2004) for the analysis of DTI images guarantee the homogeneity of the results in the feature manifold as well as the smoothness of the segmentation in the image domain. They are thus particularly interesting in our application. However, the complex structure of the DT manifold hampers the derivation of a statistically well-founded likelihood. We propose in the next paragraph to approximate, using the Martin distance, the definition of the intrinsic normal distribution on Riemannian manifolds presented by Pennec (2006) and to derive our motion-segmentation likelihood from this approximation.

#### 2.2.1 Approximation of the normal distribution on the DT manifold

We assume a normal distribution for the DT models in each region. The *pdf* of a normal distribution of mean $\overset{¯}{x}$ and of concentration matrix *Γ* on a Riemannian manifold M has been defined by Pennec (2006):

(4) $$N(\overset{¯}{x}\text{,}\Gamma )(y)\propto \exp\left(-\frac{\log_{\overset{¯}{x}}(y)\Gamma \log_{\overset{¯}{x}}{\left(y\right)}^{⊤}}{2}\right)\text{,}$$

where $\log_{\overset{¯}{x}}(y)$ denotes the mapping of **y** to the tangent plane of M at $\overset{¯}{x}$. The term in the exponential can be interpreted as the distance between the projections of **y** and of $\overset{¯}{x}$ to the plane tangent to the manifold at $\overset{¯}{x}$. The concentration matrix *Γ* aims at respecting the curvature of the manifold and accounts for the dispersion of the DT models. This formulation requires the definition of the logarithm operation and of Riemannian metrics. While these quantities are well known on some manifolds such as the manifold of definite positive matrices (Lenglet et al., 2004; Pennec et al., 2006) or for the group of diffeomorphisms (Arsigny et al., 2006), they remain difficult to compute for the manifold of the DT models (C, 2010). We thus propose to approximate the projection step and the computation of the distance in the DT manifold with the previously defined Martin distance. We end up with the following approximation of Eq. (4):

(5) $$N\left({\overset{¯}{D}}_{\Omega_{k}^{t}}\text{,}\sigma_{\Omega_{k}^{t}}^{2}\right)\left(D_{x}^{t}\right)\propto \frac{1}{\sigma_{\Omega_{k}^{t}}}\exp\left(-\frac{d_{M}^{2}\left({\overset{¯}{D}}_{\Omega_{k}^{t}}\text{,}D_{x}^{t}\right)}{2\sigma_{\Omega_{k}^{t}}^{2}}\right)\text{,}$$

where ${\overset{¯}{D}}_{\Omega_{k}^{t}}$ and $\sigma_{\Omega_{k}^{t}}^{2}$ respectively denote the mean and the variance of the DT models observed in the *k*th region and at the temporal window $S^{t}$. We finally assume the independence between the labellings of the regions and the independence of the DT models in each pixel. This leads to the following expression for the likelihood energy of $S^{t}$ given *ω*^*t*^:

(6) $$E_{\mathrm{ms}}(S^{t}|\omega^{t})=\overset{3}{\underset{k=1}{\sum }}\underset{x\in \Omega_{k}^{t}}{\sum }\left[\frac{d_{M}^{2}\left(D_{x}^{t}\text{,}{\overset{¯}{D}}_{\Omega_{k}^{t}}\right)}{2\sigma_{\Omega_{k}^{t}}^{2}}+\log\left(\sigma_{\Omega_{k}^{t}}\right)\right]\text{.}$$

#### 2.2.2 Mean and variance

The above approximation of the likelihood energy relies on the computation of the mean and variance of the DT models. The common definition of the mean by the expectation, which holds for vector spaces, is not valid on non-Euclidean manifolds because of the difficulty to extend the sum with value on the manifold. The mean in the sense of Fréchet is alternatively defined as the point in the manifold that minimizes the variance to the observed points (Pennec, 2006). In our case, the Fréchet mean of the DT models observed in the region $\Omega_{k}^{t}$ reads as:

(7) $${\overset{¯}{D}}_{\Omega_{k}^{t}}=\arg\underset{D_{x}^{t}\in M}{\min}\left[\underset{y\in \Omega_{k}^{t}}{\sum }p\left(D_{y}^{t}\right)\mathrm{dist}{\left(D_{x}^{t}\text{,}D_{y}^{t}\right)}^{2}\right]\text{,}$$

where $\mathrm{dist}\left(D_{x}^{t}\text{,}D_{y}^{t}\right)$ is the geodesic distance between the DT model $D_{x}^{t}$ in the manifold M and the DT model $D_{y}^{t}$ observed in the region $\Omega_{k}^{t}$. $p\left(D_{y}^{t}\right)$ is the probability of the observed DT model $D_{y}^{t}$. Pennec (2006) proposes to solve this minimization problem by a gradient descent scheme. The computation of the new mean estimate in the tangent space of the current mean estimate is alternated with the recentring of the tangent space at the point on the manifold that corresponds to the new mean estimate. In this work, we reduce the space of search of the minimization by approximating the manifold M of the DT models by the finite set of DT models observed in the scene, i.e. $M=\left\{D_{x}^{t}|x\in \Omega^{t}\right\}$. Furthermore, we limit the number of distances to be computed by only estimating the barycenter on a sparse set $\Omega_{k\text{,}s}^{t}$ of samples randomly chosen in $\Omega_{k}^{t}$. We finally make the same approximation as above that the structure of the manifold of the DT models can be characterized by the Martin distance. These simplifications result in the tractability of the following exhaustive search:

(8) $${\overset{¯}{D}}_{\Omega_{k}^{t}}=\arg\underset{D_{x}^{t}\text{,}x\in \Omega }{\min}\left[\underset{y\in \Omega_{k\text{,}s}^{t}}{\sum }p\left(D_{y}^{t}\right)d_{M}{\left(D_{x}^{t}\text{,}D_{y}^{t}\right)}^{2}\right]\text{,}$$

for $\left|\Omega_{k\text{,}s}^{t}|\ll |\Omega_{k}^{t}\right|$.

An alternative solution to the computation of the Fréchet mean consists of approximating the squared distance between the fixed DT model $D_{x}^{t}$ and the mean DT model ${\overset{¯}{D}}_{\Omega_{k}^{t}}$ by the expectation of the squared distance between $D_{x}^{t}$ and a random DT model $D_{Y}^{t}$ representative of the region. This defines the upper bound of the mean according to Doss (Doss, 1949; Pennec, 2006). Again, the expression is made computationally tractable by restricting the summation space to the subset of random samples $\Omega_{k\text{,}s}^{t}$:

(9) $$d_{M}{\left(D_{x}^{t}\text{,}{\overset{¯}{D}}_{\Omega_{k}^{t}}\right)}^{2}=\underset{y\in \Omega_{k\text{,}s}^{t}}{\sum }p\left(D_{y}^{t}\right)d_{M}{\left(D_{x}^{t}\text{,}D_{y}^{t}\right)}^{2}\text{.}$$

The variance $\sigma_{\Omega_{k}^{t}}^{2}$ of the DT models in the region $\Omega_{k}^{t}$ can be directly computed after estimation of the mean DT models using Eq. (8) or of the squared Martin distances using Eq. (9):

(10) $$\sigma_{\Omega_{k}^{t}}^{2}=\underset{x\in \Omega_{k}^{t}}{\sum }p\left(D_{x}^{t}\right)d_{M}{\left(D_{x}^{t}\text{,}{\overset{¯}{D}}_{\Omega_{k}^{t}}\right)}^{2}\text{.}$$

A limitation of using a patch-based approach for the segmentation of DT models is that, for any patch overlapping two distinct regions, the intensities are generated by two independent processes. In this case, the estimated DT model does not correspond to the model of any region in the scene. This results in a likelihood discontinuous at the borders of the regions. Assuming that the segmented region $\Omega_{k}^{t}$ at the current iteration is a good estimate of the real position of the *k*th region, the DT models $D_{y}^{t}$ which are the more likely to correspond to a single motion pattern are the DT models whose pixels $y\in \Omega_{k}^{t}$ is the further from the borders of the object. We favor these DT models in the above estimation of the mean and of the standard deviation by setting:

(11) $$p\left(D_{y}^{t}\right)=p\left(D_{y}^{t}|y\text{,}\Omega^{t}\right)=\min\left(d_{S}{\left(y\text{,}\Omega_{k}^{t}\right)}^{2}\text{,}m^{2}\right)\text{,}$$

where $d_{S}(.\text{,}\Omega_{k}^{t})$ is the signed distance function to the region $\Omega_{k}^{t}$. This confidence value is incorporated into Eqs. (8) and (9) after normalization on the pixel samples in $\Omega_{k\text{,}s}^{t}$, and into Eq. (10) after normalization on the pixel samples in $\Omega_{k}^{t}$.

#### 2.2.3 Alternative solutions

**Fig. 3:**
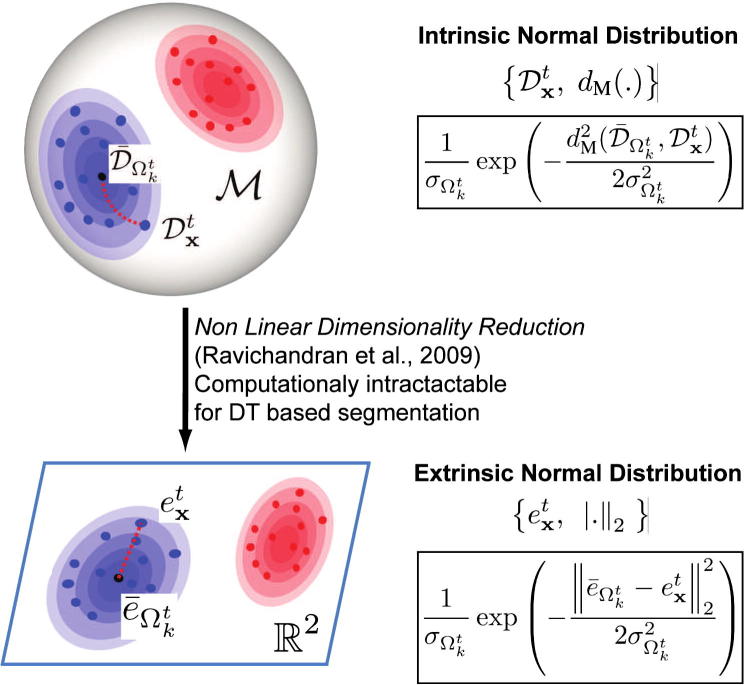
Intrinsic vs. extrinsic definition of the normal distribution. We propose to approximate the normal distribution on the manifold of the DT models using the Martin distance. An extrinsic approach, which consists of projecting the DT models into a lower dimensional Euclidean space using non-linear dimensionality reduction methods (Ravichandran et al., 2009), is intractable in our scenario because of the high number of DT models considered in patch-based segmentation problems.

In the two last paragraphs, we have introduced an approximation for the *intrinsic* normal distribution on the manifold of the DT models. Ravichandran et al. (2009) propose to solve the non-linearity problem by projecting the DT models on a low dimensional and Euclidean subspace using non-linear dimensionality reduction (NLDR) methods (cf. Fig. 3). The clustering of the DT models is performed on the Euclidean space, i.e. *extrinsically* to the manifold. Since only a few DT models are considered, the number of pairwise distances necessary to built the similarity graph is limited and the NLDR step remains computationally tractable (Ravichandran et al., 2009). This is not the case in our scenario. Given that a DT model is associated to each pixel in *Ω* (∣*Ω*∣ ≈ 2.5 × 10^4^), building the similarity graph would require the computation of ∣*Ω*∣^2^ Martin distances and would become computationally intractable. At the opposite, our approximations of the normal *pdf* and of the mean DT model make possible the reduction of the number of Martin distances which need to be estimated (∣*Ω*∣ × ∣*Ω*_*s*_∣ with ∣*Ω*_*s*_∣ ≪ ∣*Ω*∣).

### 2.3 Event-detection likelihood energy

The aim of the event-detection energy is to provide additional features, based on the variation of the DT models in time, for the segmentation of the thrombus region.

**Fig. 4:**
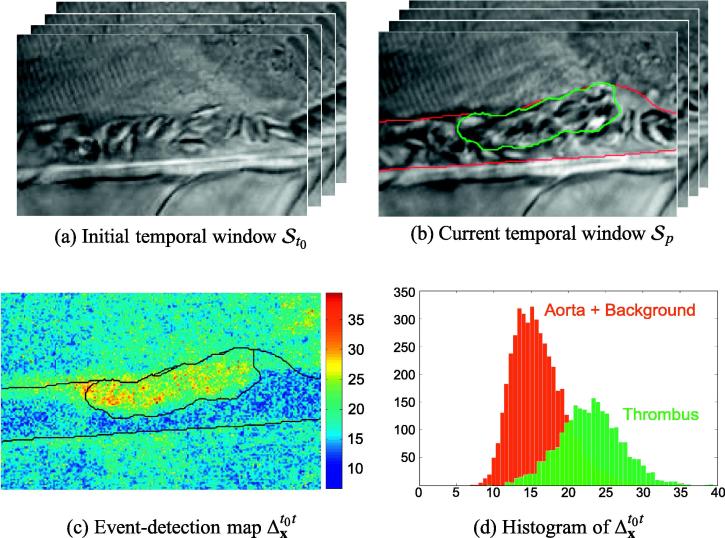
DIC microscopic images before (a) and after the formation (b) of the thrombus. (c) and (d) The study of the variation of the motion patterns between these two states leads to an additional discriminative feature for the thrombus.

In addition to its distinct motion pattern, the thrombus is characterized by its growth in an initially uncoagulated vessel. As illustrated in Fig. 4, we exploit this observation by analyzing the variations of the motion patterns between the initial temporal window $S^{t_{0}}$, in which the thrombus has not yet developed, and the current temporal window $S^{t}$.

Let us consider a pixel **x** belonging to the thrombus region at the current temporal window $S^{t}$. Since the thrombus is not developed in the first temporal window $S^{t_{0}}\text{,}\;x$ initially belongs to the background or to the uncoagulated aorta. Its motion pattern is modified and the Martin distance between the DT model $D_{x}^{t_{0}}$ observed in $S^{t_{0}}$ and the DT model $D_{x}^{t}$ observed in $S^{t}$ becomes significant. Let us consider a pixel **x** belonging to the uncoagulated vessel or to the background region in $S^{t}$. Following the observation that the vessel boundaries remain nearly constant over the video sequence, **x** belongs to the same region in $S^{t_{0}}$. Its motion pattern is unchanged and the Martin distance between $D_{x}^{t_{0}}$ and $D_{x}^{t}$ tends to zero.

We define the *event-detection* map $\Delta_{x}^{t_{0}t}$ in each pixel **x** as the Martin distance between the DT models $D_{x}^{t_{0}}$ and $D_{x}^{t}$. As shown in Fig. 4c–d, $\Delta_{x}^{t_{0}t}$ is characterized by high values for the thrombus region, and can therefore be employed as an additional discriminative feature between the thrombus and the two other regions. We assume the normal distribution of this feature in each of the *k*th region. This leads to the following definition for the *event-detection* energy:

(12) $$\begin{array}{cc} & E_{\mathrm{ed}}(S^{t}\text{,}S^{t_{0}}|\omega^{t})=\overset{3}{\underset{k=1}{\sum }}\underset{x\in \Omega_{k}^{t}}{\sum }\left[\frac{{\left(\Delta_{x}^{t_{0}t}-\mu_{\Omega_{k}^{t}}^{t_{0}t}\right)}^{2}}{2{\left(\sigma_{\Omega_{k}^{t}}^{t_{0}t}\right)}^{2}}+\log\left(\sigma_{\Omega_{k}^{t}}^{t_{0}t}\right)\right]\text{,}\\ & \mathrm{with}\;\Delta_{x}^{t_{0}t}=d_{M}\left(D_{x}^{t_{0}}\text{,}D_{x}^{t}\right)\text{,} \end{array}$$

where $\mu_{\Omega_{k}^{t}}^{t_{0}t}$ and $\sigma_{\Omega_{k}^{t}}^{t_{0}t}$ denote the mean and the standard deviation of the event-detection values $\Delta_{x}^{t_{0}t}$ in the region $\Omega_{k}^{t}$.

### 2.4 Tubular shape prior energy

**Fig. 5:**
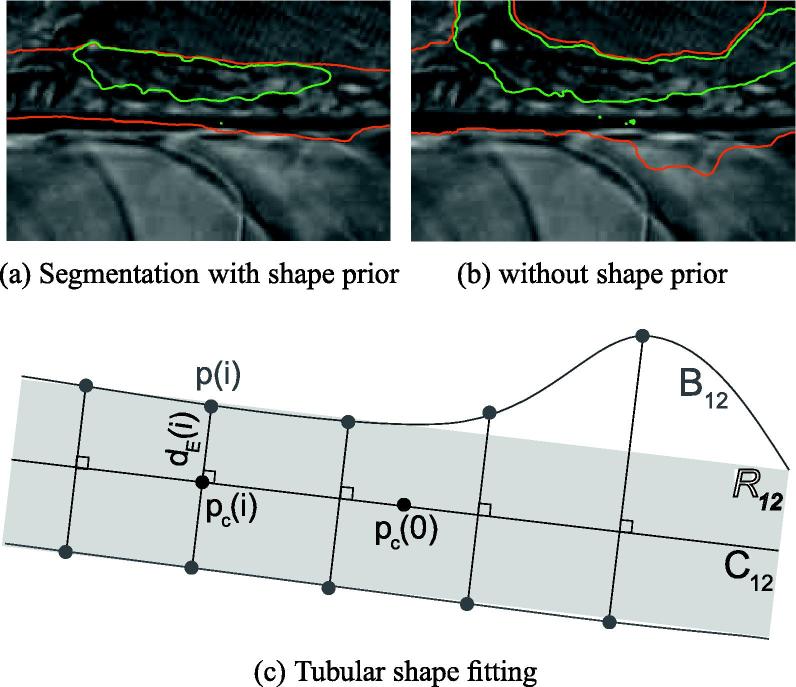
Estimated segmentation of the thrombus (green) and of the aortic vessel (red) for a sub-sequence containing collateral vessels (red stars), if the tubular shape prior on the vessel region is dismissed or incorporated. In (c), overview of our tubular shape fitting method, displaying the boundary $B_{12}$ of the vessel region $\Omega_{12}^{t}$ in dark gray, its principal axis $C_{12}$ and its center of mass *p*_*c*_(0) in black, and the reconstructed rectangle $R_{12}$ in light gray. (For interpretation of the references to colour in this figure legend, the reader is referred to the web version of this article.)

The likelihood energies defined by Eqs. (6) and (12) characterize the spatial and the temporal changes of the DT models. As illustrated in Fig. 5b, these energies and the subsequent segmentations can be occasionally disrupted by the slow circulation of blood cells in the collateral vessels and by the local non-rigid deformations of the background. These perturbations provoke leaks in the segmentation of the thrombus and of the vessel regions. To avoid such inaccuracies, we constrain the shape of the aortic region $\Omega_{12}^{t}$ with a tubular shape prior energy. At each iteration of the energy minimization scheme, we fit an “infinite rectangle” $R_{12}$ to the boundary $B_{12}$ of $\Omega_{12}^{t}$ taken at the previous iteration.

An infinite rectangle can be parameterized by its centerline $C_{12}$ and its radius *r*_12_ (cf. Fig. 5c). We approximate $C_{12}$ by the first principal axis of $\Omega_{12}^{t}$. Given *m* the slope and *p*_*c*_(0) = (*x*_*c*_(0), *y*_*c*_(0)) the center of mass of $C_{12}$, its characteristic equation reads as:

(13) C^12:yc=mxc+(yc(0)-mxc(0)).

The distance of a pixel *p*(*i*) = (*x*(*i*), *y*(*i*)) belonging to the boundary $B_{12}$ of the region to the estimated centerline C^12 is defined by the Euclidean distance ∥*p*(*i*) − *p*_*c*_(*i*)∥_2_ of this pixel to its orthogonal projection *p*_*c*_(*i*) = (*x*_*c*_(*i*), *y*_*c*_(*i*)) on C^12. The coordinates of *p*_*c*_(*i*) are obtained using basic geometry

(14) $$\left\{\begin{array}{c} x_{c}(i)=\frac{m}{1+m^{2}}(y_{c}(0)-y(i))+\frac{x(i)-m^{2}x_{c}(0)}{1+m^{2}}\\ y_{c}(i)=y(i)-\frac{1}{m}(x_{c}(0)-x(i)) \end{array}\right)\text{.}$$

We approximate the radius *r*_12_ of the fitted rectangle by the median value of the distances between the pixels on $B_{12}$ and the centerline C^12. For high angle values, the obtained parameters are further optimized using a simplex-based approach. Given the fitted rectangle $R_{12}$, we finally define the following shape prior energy:

(15) $$E_{\mathrm{sp}}(\omega^{t})=\overset{2}{\underset{k=1}{\sum }}\underset{x\in \Omega_{k}^{t}}{\sum }\left[\exp\left(\frac{d_{R_{12}}^{2}(x)}{2\sigma^{2}}\right)-1\right]\text{,}$$

where $d_{R_{12}}(.)$ denotes the distance transform of the binary image derived from the fitted rectangle *R*_12_. σ∈R sets the range of feasible deformations. This energy prevents the pixels which are far away from the fitted rectangle ($d_{R_{12}}^{2}(x)\gg \sigma^{2}$) to belong to the estimated aortic region $\Omega_{12}^{t}$.

(16) $$\nabla_{\Phi_{1}}E(\Phi_{1}\text{,}\Phi_{2}|S^{t}\text{,}S^{t_{0}})=\alpha \delta (\Phi_{1})\left[(1-H(\Phi_{2}))\left(\frac{d^{2}\left(D_{x}^{t}\text{,}{\overset{¯}{D}}_{\Omega_{2}^{t}}\right)}{2\sigma_{\Omega_{2}^{t}}^{2}}-\frac{d^{2}\left(D_{x}^{t}\text{,}{\overset{¯}{D}}_{\Omega_{1}^{t}}\right)}{2\sigma_{\Omega_{1}^{t}}^{2}}+\log\frac{\sigma_{\Omega_{2}^{t}}}{\sigma_{\Omega_{1}^{t}}}\right)+H(\Phi_{2})\left(\frac{d^{2}\left(D_{x}\text{,}{\overset{¯}{D}}_{\Omega_{3}^{t}}\right)}{2\sigma_{\Omega_{3}^{t}}^{2}}+\log\sigma_{\Omega_{3}^{t}}\right)\right]+\beta \delta (\Phi_{1})\left[(1-H(\Phi_{2}))\left(\frac{{\left(\Delta_{x}^{t_{0}t}-\mu_{\Omega_{2}^{t}}^{t_{0}t}\right)}^{2}}{2{\left(\sigma_{\Omega_{2}^{t}}^{t_{0}t}\right)}^{2}}-\frac{{\left(\Delta_{x}^{t_{0}t}-\mu_{\Omega_{1}^{t}}^{t_{0}t}\right)}^{2}}{2{\left(\sigma_{\Omega_{1}^{t}}^{t_{0}t}\right)}^{2}}+\log\frac{\sigma_{\Omega_{2}^{t}}^{t_{0}t}}{\sigma_{\Omega_{1}^{t}}^{t_{0}t}}\right)+H(\Phi_{2})\left(\frac{{\left(\Delta_{x}^{t_{0}t}-\mu_{\Omega_{3}^{t}}^{t_{0}t}\right)}^{2}}{2{\left(\sigma_{\Omega_{3}^{t}}^{t_{0}t}\right)}^{2}}+\log\sigma_{\Omega_{3}^{t}}^{t_{0}t}\right)\right]-\zeta \delta (\Phi_{1})H(\Phi_{2})$$

(17) $$\nabla_{\Phi_{2}}E(\Phi_{1}\text{,}\Phi_{2}|S^{t}\text{,}S^{t_{0}})=\alpha \delta (\Phi_{2})\left[-(1-H(\Phi_{1}))\left(\frac{d^{2}\left(D_{x}^{t}\text{,}{\overset{¯}{D}}_{\Omega_{1}^{t}}\right)}{2\sigma_{\Omega_{1}^{t}}^{2}}+\log\sigma_{\Omega_{1}^{t}}\right)+H(\Phi_{1})\left(\frac{d^{2}\left(D_{x}^{t}\text{,}{\overset{¯}{D}}_{\Omega_{3}^{t}}\right)}{2\sigma_{\Omega_{3}^{t}}^{2}}-\frac{d^{2}\left(D_{x}^{t}\text{,}{\overset{¯}{D}}_{\Omega_{2}^{t}}\right)}{2\sigma_{\Omega_{2}^{t}}^{2}}+\log\frac{\sigma_{\Omega_{3}^{t}}}{\sigma_{\Omega_{2}^{t}}}\right)\right]+\beta \delta (\Phi_{2})\left[-(1-H(\Phi_{1}))\left(\frac{{\left(\Delta_{x}^{t_{0}t}-\mu_{\Omega_{1}^{t}}^{t_{0}t}\right)}^{2}}{2{\left(\sigma_{\Omega_{1}^{t}}^{t_{0}t}\right)}^{2}}+\log\sigma_{\Omega_{1}^{t}}^{t_{0}t}\right)+H(\Phi_{1})\left(\frac{{\left(\Delta_{x}^{t_{0}t}-\mu_{\Omega_{3}^{t}}^{t_{0}t}\right)}^{2}}{2{\left(\sigma_{\Omega_{3}^{t}}^{t_{0}t}\right)}^{2}}-\frac{{\left(\Delta_{x}^{t_{0}t}-\mu_{\Omega_{2}^{t}}^{t_{0}t}\right)}^{2}}{2{\left(\sigma_{\Omega_{2}^{t}}^{t_{0}t}\right)}^{2}}+\log\frac{\sigma_{\Omega_{3}^{t}}^{t_{0}t}}{\sigma_{\Omega_{2}^{t}}^{t_{0}t}}\right)\right]-\gamma \delta (\Phi_{2})\left(\exp\left(\frac{d_{R_{12}^{2}}(x)}{2\sigma^{2}}\right)-1\right)+\zeta \delta (\Phi_{2})(1-H(\Phi_{1}))$$

### 2.5 Energy minimization

The energy minimization problem defined by Eq. (1) is solved using level-sets. Level-sets methods are particularly interesting in our application because of their numerical stability and of their ability to easily combine segmentation cues with spatial regularization and shape priors. The regions $\left\{\Omega_{1}^{t}\text{,}\Omega_{2}^{t}\text{,}\Omega_{3}^{t}\right\}$ are represented as the zero level-sets of two functions *Φ*_1_ and *Φ*_2_. Similarly to Baust and Navab (2010), we define the thrombus region $\Omega_{1}^{t}$ by the pixels inside the two contours (*Φ*_1_ < 0, *Φ*_2_ < 0), the uncoagulated vessel region $\left(\Omega_{2}^{t}\right)$ by the pixels outside the first contour and inside the second contour (*Φ*_1_ > 0, *Φ*_2_ < 0), and the background region $\left(\Omega_{3}^{t}\right)$ by the pixels outside the two contours (*Φ*_1_ > 0, *Φ*_2_ > 0). The aortic region ($\Omega_{12}^{t}$) is thus directly represented by the zero level-set of *Φ*_2_. The topological energy *E*_top_ constrains the number of regions in the partition by penalizing the surface area of the remaining and fourth region (*Φ*_1_ < 0, *Φ*_2_ > 0). Given *H* the smooth approximation of the Heaviside function, *E*_top_ reads as:

(18) $$E_{\mathrm{top}}(\Phi_{1}\text{,}\Phi_{2})=\int_{\Omega }(1-H(\Phi_{1}(x)))H(\Phi_{2}(x))dx\text{.}$$

The weighted sum of the likelihood and prior energies forms the following *a posteriori* energy:

(19) $$E(\Phi_{1}\text{,}\Phi_{2}|S^{t}\text{,}S^{t_{0}})=\alpha E_{\mathrm{ms}}(S^{t}|\Phi_{1}\text{,}\Phi_{2})+\beta E_{\mathrm{ed}}(S^{t}\text{,}S^{t_{0}}|\Phi_{1}\text{,}\Phi_{2})-\gamma E_{\mathrm{sp}}(\Phi_{1}\text{,}\Phi_{2})+\zeta E_{\mathrm{top}}(\Phi_{1}\text{,}\Phi_{2})\text{.}$$

This function is minimized by alternating until convergence an update of the energies with a diffusion-based regularized gradient descent on *Φ*_1_ and *Φ*_2_ (Baust et al., 2010):

(20) $$\begin{array}{cc} & \Phi_{1}=\Phi_{1}-\kappa \nabla_{\Phi_{1}}E(\Phi_{1}\text{,}\Phi_{2}|S^{t}\text{,}S^{t_{0}})+\lambda \mathrm{div}\left(g_{1}\nabla \Phi_{1}^{t}\right)\\ & \Phi_{2}=\Phi_{2}-\kappa \nabla_{\Phi_{2}}E(\Phi_{1}\text{,}\Phi_{2}|S^{t}\text{,}S^{t_{0}})+\lambda \mathrm{div}\left(g_{2}\nabla \Phi_{2}^{t}\right) \end{array}$$

Such Laplacian regularized level-set approaches benefit from their fast convergence and high capture range. The gradients $\nabla_{\Phi_{1}}E(\Phi_{1}\text{,}\Phi_{2}|S^{t}\text{,}S^{t_{0}})$ and $\nabla_{\Phi_{2}}E(\Phi_{1}\text{,}\Phi_{2}|S^{t}\text{,}S^{t_{0}})$ are given in Eqs. (16) and (17) respectively. To simplify the term corresponding to the event-detection energy, we assume that the distribution of the event-detection maps in the background and in the vessel follows the same normal distribution. This is qualitatively justified in Fig. 4. In Eqs. (16) and (17), *δ*(.) denotes the smooth approximation of the Dirac delta function. λ∈R is the step size. κ∈R is a parameter weighting the diffusion-based regularization. *g*_*k*_ is the diffusion coefficient of the level-set embedding function *Φ*_*k*_. It is more precisely defined by:

(21) $$g_{k}(x)=\frac{1}{1+|\nabla I^{t}{|}^{2}/\eta_{k}}\text{,}$$

where $I^{t}$ is the image to be segmented. *η*_*k*_ is the median value of the gradient magnitude of $I^{t}$ along the k^th^ contour at the current iteration. This gradient-dependent diffusion coefficient slows down the evolution of the level-sets for high values of the gradient magnitude. This yields further matching of the evolving contours to the edges of the aortic and of the thrombus regions.

## 3 Results

The first aim of this section is the validation of the segmentation algorithm presented in Section 2. Section 3.1 tests the likelihood and prior energies on synthetic examples. Section 3.2 quantifies the performance of the level-set framework on real microscopic sub-sequences. The second aim of this section is the extension of the segmentation algorithm to the tracking of the thrombus and aortic regions in time. Section 3.3 details and quantitatively test our tracking strategy. It also discusses the automatized computation of the aforementioned time to attachment (TTA) and thrombus surface area (TSA).

### 3.1 Validation on synthetic data

In this section, we test the performance of the motion-segmentation energy (Section 3.1.1), of the event-detection energy (Section 3.1.2), and of the tubular shape prior (Section 3.1.3).

#### 3.1.1 Motion segmentation likelihood energy

We test our motion-segmentation energy and multiphase level-set framework against state-of-the-art methods for dynamic texture segmentation (Chan and Vasconcelos, 2008, 2009; Doretto et al., 2003b; Ghoreyshi and Vidal, 2007) on a exhaustive dataset of 199 synthetic sequences (Chan and Vasconcelos, 2011). Evaluation on this database is particularly attractive because the results obtained with MDT (Chan and Vasconcelos, 2008) and LDT (Chan and Vasconcelos, 2009) are publicly available, that it is exhaustive in size, and that it covers a wide variety of motion patterns including grass, water, steam, fire, etc.

In this work, we focus on the segmentation of two and three dynamic textures. This is justified by the applicative context of our developments, i.e. by the partition of the image into three dynamic regions if the thrombus has developed and into two dynamic regions otherwise. In this last case, only one level-set embedding function is necessary. Please note that our approximation of the *pdf* stands for any number of dynamic regions and could therefore, in theory, be employed in more complex scenarios than the one in the focus of this work.

We discard the event-detection energy, the tubular shape prior energy, by setting *β* = 0 and *γ* = 0 respectively. This allows us to drive the segmentation only with the motion-segmentation and the topological energies. Also, and for all the experiments conducted on synthetic data, we set *η* = ∞ to smooth the level-set embedding functions independently to the possible edges of the objects.

The initial contours and the parameters of the model (*n* = 15, *m* = 5 × 5, *τ* = 60) are the same as in (Chan and Vasconcelos, 2009). The same parameters are also used for the MDT (Chan and Vasconcelos, 2008), the LDT (Chan and Vasconcelos, 2009), and the single DT (Doretto et al., 2003b) methods. The parameters of the Ising approach are taken from (Ghoreyshi and Vidal, 2007). The same level-set framework, described in Section 2.5, is used for our algorithm and for the other level-set based approaches (Doretto et al., 2003b; Ghoreyshi and Vidal, 2007) in order to ensure a fair comparison of the segmentation energies. The results obtained with MDT and LDT are directly taken from (Chan and Vasconcelos, 2008, 2009).

##### 3.1.1.1 Qualitative performance

**Fig. 6:**
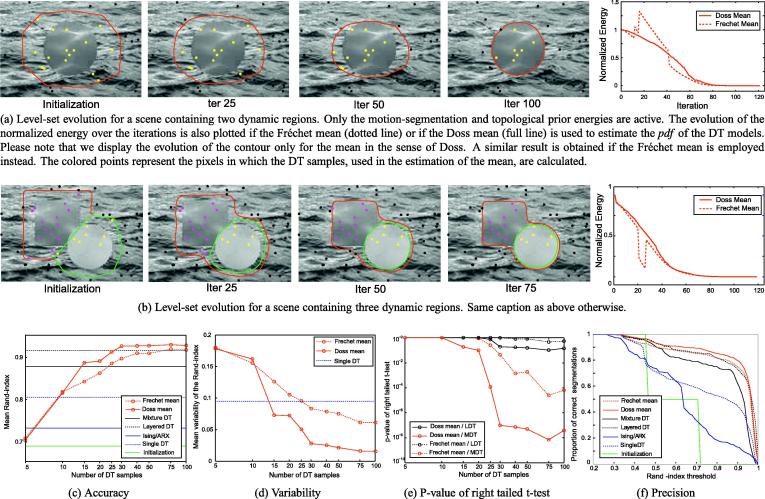
Qualitative and quantitative evaluation of the motion-segmentation likelihood, estimated using either the Fréchet mean or the mean in the sense of Doss. (a) and (b) Level-set evolution for two of the 199 synthetic sequences (Chan and Vasconcelos, 2011). (c) and (d) Accuracy and robustness of our method with respect to the selection of the random DT samples, in function of the number of DT samples and in comparison to the state-of-the-art methods (Chan and Vasconcelos, 2008, 2009; Doretto et al., 2003a; Ghoreyshi and Vidal, 2007). (e) *P*-value that the mean accuracy using our method is smaller than the mean accuracy using (Chan and Vasconcelos, 2008, 2009). (f) Precision curves obtained for ∣*Ω*^*s*^∣ = 40 DT samples.

The performance of our motion-segmentation energy is qualitatively illustrated in Fig. 6a and b on two synthetic sequences containing two and three dynamic regions respectively. For the scene with two dynamic regions, the circle is successfully segmented. We also achieve accurate segmentation of the scene with three dynamic textures. In this case, the disk models the thrombus and the square models the uncoagulated vessel. It is important to observe in the energy-minimization plots at the far right of Fig. 6a and b that the selection of a unique DT sample in the estimation of Fréchet mean results in a discontinuous segmentation-energy (dotted curve). The mean in the sense of Doss yields a smoother convergence (full curve).

##### 3.1.1.2 Quantitative performance

We estimate for each sequence the Rand-index (Hubert and Arabie, 1985) between the ground-truth segmentation and the segmentations obtained with our approach and with the state-of-the-art methods (Chan and Vasconcelos, 2008, 2009; Doretto et al., 2003b; Ghoreyshi and Vidal, 2007). Using the Rand-index ensures a fair comparison of our approach with the two most recent methods for dynamic texture segmentation (Chan and Vasconcelos, 2008, 2009). Results are presented in Fig. 6c. Our algorithm achieves a mean Rand-index higher than *r* = 0.91 ± 14 and *r* = 0.93 ± 0.12 if more than ∣*Ω*_*s*_∣ = 40 reference points are considered and that the Fréchet and the Doss means are employed respectively. This is a significant improvement in comparison to the initialization (*r* = 0.69). In comparison, the MDT approach (Chan and Vasconcelos, 2008) yields *r* = 0.88 ± 13, the LDT algorithm (Chan and Vasconcelos, 2009) *r* = 0.92 ± 0.13, the single DT approach (Doretto et al., 2003b) *r* = 0.81 ± 0.17, and the Ising/AR model (Ghoreyshi and Vidal, 2007) *r* = 0.73 ± 0.14. While using the Fréchet mean yields a similar accuracy than employing LDT, using the Doss mean leads to a significant improvement of the accuracy in comparison to all the state-of-the-art algorithms, including LDT. The higher performance of the Doss mean can be explained by the robustness of Eq. (9) with respect to noise. The noise in the Martin distance maps $d_{M}\left(D_{x}^{t}\text{,}D_{y}^{t}\right)$ tends to cancel out by addition.

It is crucial to observe that, given a fixed initialization, the approaches (Chan and Vasconcelos, 2008, 2009; Ghoreyshi and Vidal, 2007) are deterministic. At the opposite, our algorithm and the single DT method (Doretto et al., 2003b) depends on the selection of the DT samples and of the reference model respectively. The robustness with respect to the selection of the DT models is measured by the standard deviation of the Rand-indexes of the segmentations for five runs of the algorithms. As shown in Fig. 6d, the variability of our approach with respect to the selection of the DT samples stays smaller than 0.08 ± 0.09 (Fréchet) and 0.025 ± 0.05 (Doss) for $\left|\Omega_{p}^{s}\right|>40$ DT samples. As expected, the single DT approach (Doretto et al., 2003b) is characterized by a higher mean variability of *r* = 0.10 ± 0.07.

To check if the robustness of our approach with respect to the selection of the DT models and to the variety of motion patterns is sufficient to conclude on its better performance, we perform right-tailed Student t-tests between our results and the results obtained with the two most accurate state-of-the art methods, i.e. with MDT (Chan and Vasconcelos, 2008) and LDT (Chan and Vasconcelos, 2009). We show in Fig. 6e that the *p*-values with respect to MDT stay smaller than 0.01 for both the Doss and the Fréchet means if ∣*Ω*_*s*_∣ > 40 DT samples are selected. We also note that, even though there is no statistical difference between the results obtained with the Fréchet mean and the results obtained with the LDT approach (Chan and Vasconcelos, 2009) (*p* ≈ 0.5), we can successfully conclude on the better performance of our segmentation framework if the Doss mean is employed (*p* < 0.17).

We quantify the robustness of the aforementioned algorithms with respect to the variety of the motion patterns by computing the precision curves over the sequences in the database. These curves represent the proportion of segmentations having a Rand-index larger than a varying threshold. The precision curves obtained using our level-set framework and the Doss mean stays at the top-right of the precision curves obtained with (Chan and Vasconcelos, 2008, 2009; Doretto et al., 2003b; Ghoreyshi and Vidal, 2007), showing that we can correctly segment a higher proportion of dynamic texture sequences than the state-of-the-art methods (cf. Fig. 6f).

In this paragraph, we have shown experimental evidence of the performance of our motion-segmentation energy and level-set framework in comparison to state-of-the-art methods for dynamic texture segmentation. We solve the two problems posed by the original segmentation method (Doretto et al., 2003b) based on single DT model, i.e. the choice of the reference model and the incapacity of segmenting multiple dynamic regions, while maintaining the simplicity of the LDS formulation for dynamic textures and of the closed-form solution (Doretto et al., 2003a) for parameter estimation.

#### 3.1.2 Event-detection likelihood

**Fig. 7:**
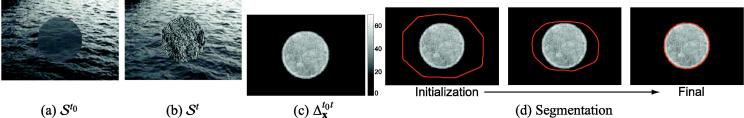
Pixel wise comparison of the motion patterns observed in the sequence (a) and in the sequence (b) using the Martin distance. (c) Resulting event-detection map $\Delta_{x}^{t_{0}t}$. (d) Level-set evolution using only our event-detection likelihood energy. Changes of the DT models are accurately segmented.

Fig. 7a and b display two synthetic sequences containing a dynamic foreground object overlaid on a dynamic background. The two sequences are taken from the synthetic database (Chan and Vasconcelos, 2011) and are temporally shifted to ensure that the intensity observed in the sequence $S^{t_{0}}$ at any pixel location differs from the intensity observed in $S^{t}$ at the same pixel location. The shape of the foreground object remains the same between the two sequences. The modification of its motion patterns models the appearance of the thrombus. We choose *α* = 0, *γ* = 0, *ζ* = 0, and *σ* = ∞ to evolve the level-set embedding function only with the event-detection energy.

We observe in Fig. 7c that the foreground region is characterized by high values in the event-detection map $\Delta_{x}^{t_{0}t}$. Fig. 7(d) shows that driving the evolution of the level-set with the event-detection energy Eq. (12) results in a successful estimation of the foreground object. We repeat the same experiment on 50 different pairs of sequences and obtain a mean rand-index of *r* = 0.94 ± 0.11 and a mean Dice-coefficient (Dice, 1945) of *DC* = 0.91 ± 0.19.

#### 3.1.3 Tubular shape prior energy

**Fig. 8:**
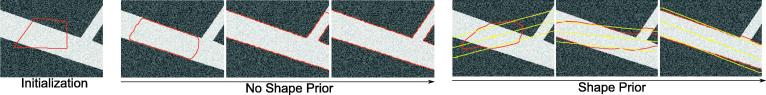
Intensity-based level-set segmentation of a synthetic model for the aortic and collateral vessels with and without tubular shape prior energy.

We qualitatively validate our shape prior energy on a synthetic model of the aortic and collateral vessels. The vessels are represented by high intensity values, the background by low intensity values. We replace our motion-segmentation energy by an intensity-based likelihood (Cremers et al., 2007). We set *β* = 0 and *ζ* = 0 to discard the event-detection and topological energies. As shown in Fig. 8, discarding the shape prior energy results in the incorporation of the collateral vessels into the segmentation result. As expected, our shape prior energy prevents the contour from leaking into the collateral vessels and leads to a more specific estimation of the synthetic aortic vessel.

### 3.2 Real microscopic data

In Section 3.1, we have shown experimental evidence of the performance of our segmentation algorithm and of each of the newly introduced energies on synthetic examples. We now test the performance of our segmentation method on real *in vivo* microscopic sequences. We start this section by detailing the experimental setup in Section 3.2.1. We then analyze the accuracy of our segmentation algorithm on a set of real microscopic sub-sequences in Section 3.2.2, and show the necessity of incorporating each of the introduced energies to obtain accurate segmentation results in Section 3.2.3. Our segmentation approach is used for the tracking of the thrombus and of the vessel and for the automatized computation of TTA and TSA in Section 3.3.

#### 3.2.1 Protocol

The zebrafish larvae are 3–4 days old. They are anesthetized in Tricaine solution (300 μM, Sigma–Aldrich). To initiate thrombus formation, the epithelium of their caudal aorta is injured using a nitrogen-pulse ablating laser (VSL-337, LSI Laser Science). The images are recorded in time with a Leica DFC420-C camera through a Differential Interference Contrast (DIC) Leica DMRXA microscope equipped with a 40 × water-immersed objective. A region of interest containing the thrombus and the aortic vessel is selected. The images are down-sampled by a factor two and rigidly registered to the first frame. The obtained field of view is of 110 μm × 70 μm for a resolution of 125 × 200 pixels. The frame rate is 25 fps and the exposure time 15 ms. Twenty sub-sequences taken from four *in vivo* microscopic video sequences are used for quantitative and qualitative validation of our segmentation algorithm. Variations between the test subsequences include differences in the size of the thrombus, in the contrast of the aggregated blood cells, and in the location of the collateral vessels. For each sub-sequence, the ground-truth segmentations are manually delineated by a trained biologist. Accuracy is measured with the Dice-coefficient (Dice, 1945), for both the thrombus (*DC*_*t*_) and aortic (*DC*_*v*_) regions. If not mentioned otherwise, the weighting and the shape prior parameters are empirically set to (*α* = 1, *β* = 0.5, *γ* = 0.1, *ζ* = 1.25, *σ* = 5), the order of the DT models to *n* = 15, and the size of the temporal window to *τ* = 75. The influence of these parameters on the segmentation results is studied in detail in Section 3.2.3. The size of the spatial patches (*m* × *m* = 5) and the number of DT samples (∣*Ω*_*s*_∣ = 60) are empirically fixed. The initial contours consist of polygonal region of interests, which are defined by the selection of three to five vertices to partially overlap the regions to be segmented. This last constraint arises from our choice of a level-set based framework. Please note that we employ a single initialization per sub-sequence in Sections 3.2.2 and 3.2.3. The robustness of our approach towards the selection of the initial contours is studied in Section 3.2.4.

#### 3.2.2 Performance

**Fig. 9:**
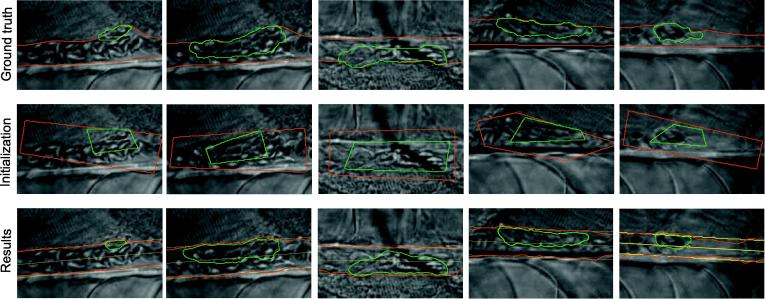
Qualitative performance of our segmentation algorithm. Montage of 5 of the test sub-sequences. The boundaries are green for the thrombus and red for the aorta. (a) Ground-truth segmentations. (b) Initial contours. (c) Segmentation results. The yellow circle and lines indicate the center *p*_*c*_(0) and the boundaries $B_{12}$ of the fitted rectangle respectively.

Fig. 9 qualitatively shows the accuracy of the segmentations obtained with our algorithm. The initial contours are characterized by an overall overlap of *DC*_*t*_ = 0.66 for the thrombus plug and of *DC*_*v*_ = 0.69 for the aortic region. In comparison, our algorithm achieves mean accuracies of *DC*_*t*_ = 0.75 and of *DC*_*v*_ = 0.88 for the aorta and for the thrombus if the Doss mean is used, and mean accuracies of *DC*_*t*_ = 0.74 and of *DC*_*v*_ = 0.86 if the Fréchet mean is employed. These values demonstrate the ability of our method to achieve accurate segmentation results despite challenging imaging conditions and far-away initializations. In our scenario, the good convergence of our algorithm is crucial to ensure that the contour follow the thrombus and the vessel regions over the sequence even in the case of fast changes of their surface area. Please note that overlap-values also confirm the better performance of the mean in the sense of Doss in comparison to the Fréchet mean (cf. Section 3.1.1 on synthetic examples). They also illustrate the difficulty of segmenting the thrombus in comparison to the aortic region. Specific challenges include its restricted surface area and the possible changes of its boundaries during the considered sub-sequences. This last point breaks the stationarity assumption inherent to the dynamic texture model and results in the temporal smoothing of the estimated thrombus region.

#### 3.2.3 Parameter setting

**Fig. 10:**
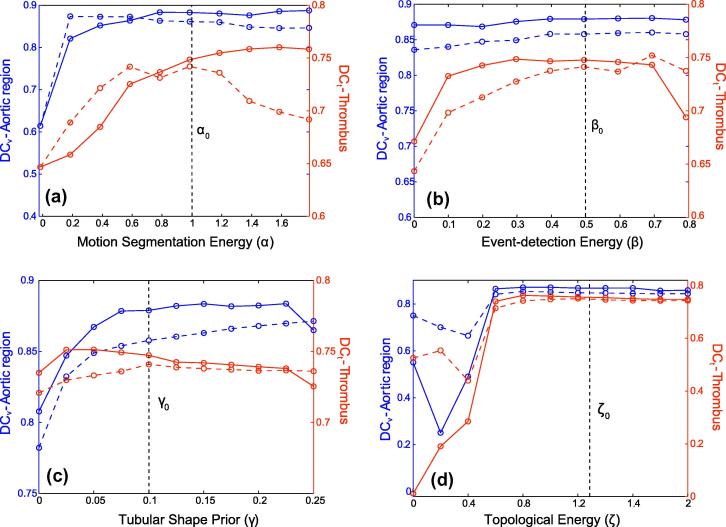
Accuracy of the segmented thrombus (in red) and of the segmented aortic region (in blue) function of the weighting parameters-*α* (a), *β* (b), *γ* (c), and *ζ* (d). (For interpretation of the references to colour in this figure legend, the reader is referred to the web version of this article.)

**Fig. 11:**
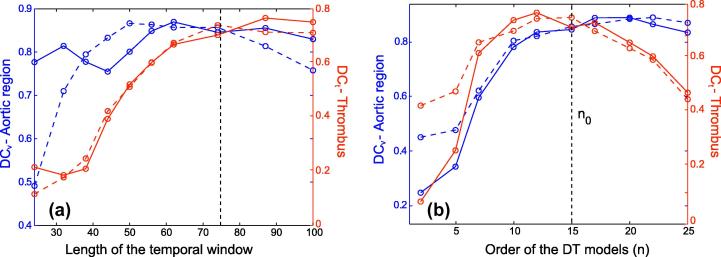
Accuracy of the segmented thrombus (in red) and of the segmented aortic region (in blue) function of the length of the temporal windows *τ* (a) and of the order of the DT model *n* (b). (For interpretation of the references to colour in this figure legend, the reader is referred to the web version of this article.)

The dependency of the results with respect to the selection of the weighting parameters and of the dimensions of the DT models is analyzed in Figs. 10 and 11. Each parameter is independently studied. The remaining parameters are set to their aforementioned values.

– If the motion-segmentation energy is discarded (*α* = 0), the Dice coefficients fall down to *DC*_*t*_ = 0.61 and *DC*_*v*_ = 0.65.
– If the event-detection energy is discarded (*β* = 0), the accuracy of the estimated thrombus region decreases to *DC*_*t*_ = 0.67 for the Doss mean and to *DC*_*t*_ = 0.64 for the Fréchet mean instead of *DC*_*t*_ = 0.75 and *DC*_*t*_ = 0.74 for *β* = 0.5 respectively.
– If the shape prior energy is discarded (*γ* = 0), the accuracy of the estimated aortic region decreases to *DC*_*v*_ = 0.81 for the Doss mean and to *DC*_*t*_ = 0.78 for the Fréchet mean instead of *DC*_*v*_ = 0.88 and *DC*_*v*_ = 0.86 for *γ* = 0.5 respectively.
– If the topological energy is discarded (*ζ* = 0), the accuracy of the segmentations drops to less than *DC*_*t*_ = 0.52 and *DC*_*v*_ = 0.76.

With respect to the dimension of the DT models, and as indicated by Fig. 11, a minimum sub-sequence length of *τ* = 75 frames and a minimal order of *n* = 10 are necessary to achieve acceptable segmentation results. We also observe that the accuracy of the thrombus segmentation falls if the order of the DT-model exceeds *n* = 15. This can be explained as follows. The estimation of the state-transition matrix A, detailed in Section 2.1, corresponds to a least-square problem with *n* ×  (*τ* − 1) equations and *n*^2^ unknowns. The higher *n* is, the less overdetermined the system becomes and the less accurate the estimate of A is. There is thus a trade-off between the characterization of the motion patterns and the accurate estimation of the models.

#### 3.2.4 Robustness towards initialization

**Fig. 12:**
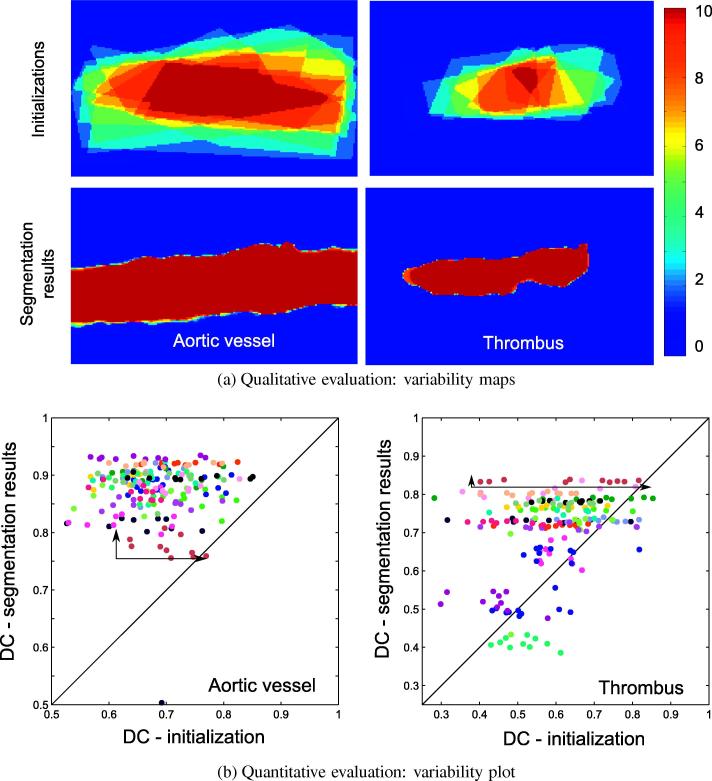
Variability of the segmentation accuracy function of the quality of the initializations for the thrombus plug and the aortic region. (a) Qualitative evaluation on the subsequence displayed in Fig. 9, column 2. The variability map shows for each pixel its frequency of inclusion in the 10 initial (top) or final (bottom) segmentations. In this case, *Σ*^Init^ = 0.15 and *Σ*^Seg^ = 0.99 for the thrombus and *Σ*^Init^ = 0.74 and *Σ*^Seg^ = 0.98 for the aortic vessel. (b) Dice coefficients final segmentations function of the Dice coefficients of the initializations. A different color is associated to each test subsequence.

We analyze the robustness of our segmentation algorithm with respect to the selection of the initial contours. For each subsequence, we select *n*_*i*_ = 10 different initial configurations and run our segmentation algorithm. We then define the initial *cross stability Σ*^Init^ as the mean of the pairwise DC values between the ten initializations, which reads as

(22) $$\Sigma^{\mathrm{Init}}=\frac{1}{n_{i}^{2}}\overset{n_{i}}{\underset{p=1}{\sum }}\overset{n_{i}}{\underset{q=1}{\sum }}\mathrm{DC}(\mathrm{Init}(p)\text{,}\mathrm{Init}(q))\text{,}$$

and the output *cross stability Σ*^Seg^ as the mean of the pairwise DC values between the ten associated segmentation results. The *cross stability* tends to one if the segmentations overlap one with each other and to 0 otherwise. This is more intuitively illustrated in Fig. 12a on a test subsequence. Over all the test subsequences, we obtain $\Sigma_{t}^{\mathrm{Init}}=0.43\pm 0.34$ and $\Sigma_{v}^{\mathrm{Init}}=0.77\pm 0.11$ between the initializations of the thrombus and aortic regions and $\Sigma_{t}^{\mathrm{Seg}}=0.96\pm 0.11$ and $\Sigma_{v}^{\mathrm{Seg}}=0.97\pm 0.05$ between the segmentation results. This increase shows the stability of our algorithm with respect to an unstable selection of the initial contours. We additionally plot in Fig. 3.2.3 the Dice coefficients, with respect to the ground truth segmentations, of the segmentation results vs the initial segmentations. The dispersion of the DC values in the *x*-axis direction, corresponding to the initialization, exceeds the of the DC values dispersion in the y-axis direction, corresponding to the obtained segmentations, for most of the test subsequences. This shows the decrease in the variability of the accuracies with respect to the ground-truth segmentations. Please note that the light-blue points are obtained on a subsequence in which the small size of the thrombus challenges the segmentation. More globally, the good stability of our approach with respect to the initialization is explained by our choice of region-based energies and by the use of a regularized gradient-descent scheme for the evolution of the two level-set functions (Baust et al., 2010).

In this section, we have shown the ability of the proposed algorithm to segment microscopic scenes under low contrast and highly dynamic conditions. In addition, we have quantitatively demonstrated the necessity to incorporate the motion-segmentation, event-detection, shape prior, and topological energies into the level-set framework as well as the robustness of our method with respect to the choice of the initial contours. The determination of quantitative measures such as the time to attachment and the thrombus surface area leads to the extension of the presented segmentation framework to the tracking of the vessel and of the thrombus in time. This is the focus of the Section 3.3.

### 3.3 Tracking

#### 3.3.1 Tracking strategy

We perform the tracking by initializing the level-set embedding functions at a given frame using the already segmented frames. In this case, a single vertex-based initialization becomes necessary to segment the whole video sequence.

The late development of the thrombus hampers the initialization of the function *ϕ*_1_ corresponding to the thrombus region in the first temporal window. Therefore, we initialize *Φ*_1_ and *Φ*_2_ in the last temporal window, in which the thrombus has already developed. We then propagate the segmentation results in the backward direction. The segmentation results obtained for the frame $I^{t+1}$ is used as initialization for the segmentation of $I^{t}$. Because of the minimal changes of the vessel boundaries in comparison to the thrombus plug, one can additionally regularize the temporal variation of the vessel region by introducing a term $\epsilon \delta (\Phi_{2})\left(1-2H\left(\phi_{2}^{t+1}\right)\right)$ to the gradient of the energy associated to the second level-set, where $\phi_{2}^{t-1}$ is the level-set embedding function of the output segmentation at time *t* + 1. There is a balance between the benefits of such regularization, such as temporal smoothing, and the necessity to be robust to a possible false segmentation at *t* + 1 and, as such, to minimize the propagation of error from *t* + 1 to *t*. This leads us to choose a small value for *ϵ* = 10^−2^ in comparison to the other weighting parameters.

Because of the high number of frames in the video sequences (*T* > 1000), we lower the actual temporal resolution of the method from 25 fps to 2.5 fps. The segmentations at the remaining frames are computed using shape-based interpolation (Herman et al., 1992). Also, because of the higher performance of the mean in the sense of Doss in comparison to the Frćhet mean, only the Doss mean is employed for tracking.

The performance of our algorithm is quantified against manually delineated ground-truth segmentations, available at every 15 frame of the video sequence (1.6 fps). The temporal shift between the ground-truth segmentations and the “actively” segmented frames (by opposition to the segmentations obtained by interpolation) allows us to incorporate the interpolation step in the estimation of the accuracy.

#### 3.3.2 Results and discussion

**Fig. 13:**
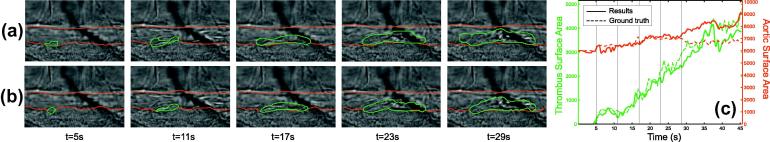
Tracking of the thrombus (in green) and vessel (in red) regions in the sequence Seq. #1. (a) Ground-truth segmentation. (b) Results obtained using our segmentation/tracking framework. (c) Surface areas of the thrombus and of vessel for both the ground truth and the estimated segmentations. (For interpretation of the references to colour in this figure legend, the reader is referred to the web version of this article.)

**Fig. 14:**
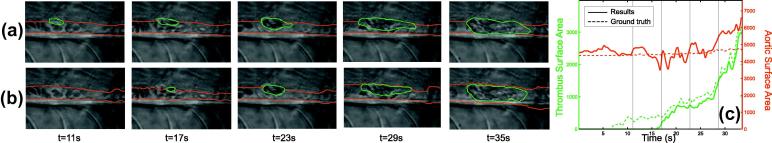
Tracking of the thrombus and vessel regions in the sequence Seq. #2. Same caption as above otherwise.

**Fig. 15:**
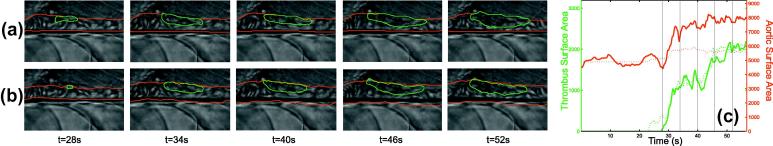
Tracking of the thrombus and vessel regions in the sequence Seq. #3. Same caption as above otherwise.

**Fig. 16:**
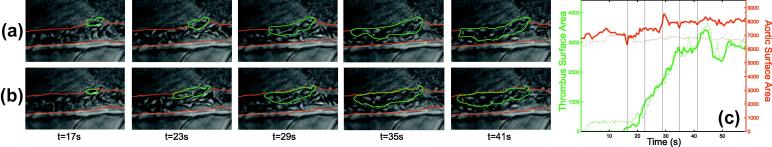
Tracking of the thrombus and vessel regions in the sequence Seq. #4. Same caption as above otherwise.

We test the performance of the tracking strategy on four microscopic video-sequences. We refer the reader to Figs. 13–16 and to Tables 1 and 2 for qualitative and quantitative results. In the two following paragraphs, we discuss the ability of our algorithm to compute biological measures such as the time to attachment (TTA) and the thrombus surface area (TSA). It is important to note that the bottle neck of a manual analysis is the computation of the TSA in time. TTA does not require the manual annotation of the images and is as such relatively easy and quick to determine visually.

We achieve a mean detection rate of 0.86 ± 0.12 for the thrombus region, and an average absolute error of 6.75 ± 5.1 s for TTA. This lack of sensitivity for early-state thrombus can be explained as follows. In Seq.#4, the local and non-rigid deformations of the background before the attachment of the first blood cell at *t* = 2 s, and the deformation of the aortic vessel by the first attaching blood cells contradict the assumptions of the event-detection and shape prior energies. This leads to the classification of the first blood cells in the background region. In Seq.#2, the thrombus is out of focus and overlaid with circulating blood cells. The motion patterns of the circulating blood cells disrupt the estimation of the DT models and the computation of the DT-based likelihoods. It therefore seems difficult to automatize the computation of the TTA.

However, and as mentioned above, the estimation of the TTA is not the bottle neck of a manual processing. Estimating the temporal variations of the thrombus surface area (TSA) requires the manual delineation of the thrombus in each frame of the sequence. The ability of the presented algorithm to accurately recover this quantity is quantified using the normalized sum of absolute differences (SADs) between the estimated and the ground truth TSAs (cf. Table 2). The normalization by the sum of the ground-truth TSAs allows an intuitive interpretation of the obtained values: the SAD tends to zero if the difference between the estimated and the ground-truth TSAs is small in comparison to the actual TSA while a value higher than one indicates an error higher than the actual TSA. We obtain a normalized SAD of 0.15 ± 0.07, which quantitatively confirms the ability of our approach to correctly estimate the TSA.

We also observe in Tables 1 and 2 that switching one of the energies off yields a decrease in accuracy and robustness of the detection rate, mean Dice coefficient, TSA, and TTA. This shows the importance of incorporating each of the energies into the tracking/segmentation framework and as such confirms the results of Section 3.2.3 and Fig. 10.

More generally, we have shown in this section the ability of our complete framework to segment the thrombus and the aortic regions in time and to compute an accurate estimate of the temporal variations of the thrombus surface area (TSA) despite the poor *in vivo* imaging conditions.

## 4 Conclusion

In this paper, we present an image-based algorithm to the automatized characterization of thrombus formation in DIC-microscopic image sequence. We more precisely tackle the sub-problem of the joint segmentation of the thrombus and aortic regions. The main originality of our algorithm is to analyze, using the concept of dynamic textures, the discrepancies of the motion patterns in space and in time. We introduce a novel motion-segmentation likelihood to guarantee the homogeneity of the motion patterns in the thrombus, in the vessel, and in the background. We develop an event-detection likelihood which quantifies the temporal changes of the motion patterns and models the growth of the thrombus from an empty aortic vessel. These energy functions successfully compensate for the low contrast and the high dynamic conditions of *in vivo* microscopic scenes. Furthermore, to prevent the segmentations of the thrombus and of the aortic vessel from leaking into the collateral vessels, we introduce shape and topological priors. These priors respectively constrain the rectangular shape of the aortic vessel and the inclusion of the thrombus in the aortic vessel. In a final step, we extend our segmentation method to the tracking of the thrombus and vessel region in time and to the automatized estimation of the temporal variations of the thrombus surface area. We test the validity of the introduced energies and the performance of our segmentation approach on synthetic examples as well as on real *in vivo* microscopic image sequences. The ability of our image-based solution to retrieve the surface area of the thrombus opens up the way for an automatized characterization of thrombus formation in the context of large-scale biological studies.

## Figures and Tables

**Table 1:** Tracking on microscopic image sequences. Proportion of correct detections and mean Dice-coefficient after detection for the thrombus and aortic regions. Empty cells indicate that the contours already vanish while segmenting the last frame of the sequence, therefore preventing their propagation to the previous frames.

Seq.	Region	Complete version		*α* = 0		*β* = 0		*γ* = 0		*ζ* = 0	
		Detect.	DC	Detect.	DC	Detect.	DC	Detect.	DC	Detect.	DC
#1	Aorta	1.00	0.93	0.50	0.59	0.03	0.70	0.03	0.63	–	–
	Thrombus	0.99	0.73	0.60	0.71	0.13	0.70	0.40	0.47	–	–
#2	Aorta	1.00	0.92	0.18	0.78	1.00	0.91	1.00	0.45	0.027	0.27
	Thrombus	0.73	0.80	0.38	0.84	0.49	0.63	0.75	0.78	0.07	0.33
#3	Aorta	1.00	0.89	0.13	0.61	1.00	0.87	0.23	0.68	0.13	0.38
	Thrombus	0.93	0.82	0.54	0.61	0.68	0.71	0.60	0.68	0.58	0.04
#4	Aorta	1.00	0.91	0.51	0.56	–	–	1.00	0.84	–	–
	Thrombus	0.77	0.78	0.54	0.78	–	–	0.97	0.60	–	–

Mean	Aorta	1.00 ± 0.00	0.91 ± 0.02	0.33 ± 0.20	0.64 ± 0.10	0.68 ± 0.56	0.83 ± 0.11	0.57 ± 0.51	0.65 ± 0.16	0.07 ± 0.07	0.30 ± 0.04
	Thrombus	0.86 ± 0.12	0.78 ± 0.04	0.52 ± 0.09	0.74 ± 0.10	0.4 ± 0.33	0.68 ± 0.04	0.68 ± 0.24	0.63 ± 0.13	0.33 ± 0.36	0.19 ± 0.21

**Table 2:** Absolute error between the estimated and the ground-truth TTAs for the complete and degraded versions of the algorithm, and normalized SAD between the estimated and the ground-truth TSAs.

Seq.	∣TTA_gt_ − TTA_est_∣ (in sec.)					Seq.	$\sum_{t}|{\mathrm{TSA}}_{\mathrm{gt}}(t)-{\mathrm{TSA}}_{\mathrm{est}}(t)|/\sum_{t}{\mathrm{TSA}}_{\mathrm{gt}}(t)$				
	Complete version	*α* = 0	*β* = 0	*γ* = 0	*ζ* = 0		Complete version	*α* = 0	*β* = 0	*γ* = 0	*ζ* = 0
#1	0.6	18	39.0	27.0	–	#1	0.12	0.50	0.96	2.07	–
#2	9.0	20.4	16.8	8.4	24.0	#2	0.25	0.59	0.62	0.19	2.40
#3	4.2	25.8	18.0	22.8	23.4	#3	0.11	0.76	0.47	2.53	4.97
#4	13.2	27.0	–	1.8	55.8	#4	0.11	0.27	–	0.89	–

Mean	6.75 ± 5.51	22.8 ± 4.3	24.6 ± 12.5	15.0 ± 11.9	34.4 ± 18.5	Mean	0.15 ± 0.07	0.53 ± 0.25	0.68 ± 0.25	1.42 ± 1.07	3.69 ± 1.82
